# Combining Results From Multiple Evaluations of the Same Measurand

**DOI:** 10.6028/jres.116.023

**Published:** 2011-12-11

**Authors:** Rüdiger Kessel, Raghu N. Kacker, Klaus-Dieter Sommer

**Affiliations:** National Institute of Standards and Technology, Gaithersburg, MD 20899-8910 USA; Physikalisch-Technische Bundesanstalt, Braunschweig D-38116 Germany

**Keywords:** consensus value, inter-laboratory evaluations, uncertainty in measurement

## Abstract

According to the Guide to the Expression of Uncertainty in Measurement (GUM), a result of measurement consists of a measured value together with its associated standard uncertainty. The measured value and the standard uncertainty are interpreted as the expected value and the standard deviation of a state-of-knowledge probability distribution attributed to the measurand. We discuss the term metrological compatibility introduced by the International Vocabulary of Metrology, third edition (VIM3) for lack of significant differences between two or more results of measurement for the same measurand. Sometimes a combined result of measurement from multiple evaluations of the same measurand is needed. We propose an approach for determining a combined result which is metrologically compatible with the contributing results.

## 1. Introduction

A function of various calibration laboratories, measurement standards organizations, national metrol-ogy institutes (NMIs), and international organizations such as the International Bureau of Weights and Measures (BIPM), the International Organization for Standardization (ISO), the International Organization of Legal Metrology (OIML), and the International Electro-technical Commission (IEC) is to ensure that the differences are insignificant between different measured values for the same measurand determined in various places, at various times, and by various measurement procedures. Without this assurance, the world’s commerce, trade, manufacturing, engineering, and scientific research would be chaotic.

The old-time thinking concerning the uncertainty in measurement based on statistical error analysis are inappropriate for the rapidly advancing science and technology of measurement. Therefore the world’s leading authorities in metrology developed a new concept of uncertainty in measurement. This concept is described in the Guide to the Expression of Uncertainty in Measurement (GUM) [1] and extended in the International Vocabulary of Metrology, third edition (VIM3) [2]. In accordance with the GUM and the VIM3, a result of measurement is generally expressed as a pair of values: a measured quantity value and its associated standard uncertainty. The measured value and the standard uncertainty together represent a range of values being attributed to the measurand [2, Sec. 2.9]. Suppose [*x*_1_, *u*(*x*_1_)], …, [*x_n_*, *u*(*x_n_*)] are *n* different results of measurement for a common measurand believed to be sufficiently stable, where *x*_1_, …, *x_n_* are the measured values and *u*(*x*_1_), …, *u*(*x_n_*) are the corresponding standard uncertainties. In the GUM concept of uncertainty, a measured value *x_i_* and its associated standard uncertainty *u*(*x_i_*) are regarded, respectively, as the expected value and the standard deviation of an incompletely determined state-of-knowledge probability density function (pdf) attributed to the common measurand, for *i* = 1, 2, …, *n* [1].

**Fig. 1:**
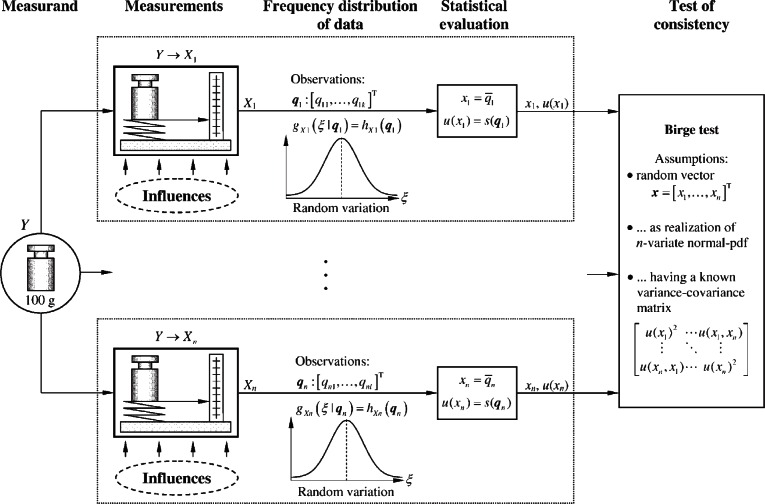
Illustration of the classical approach to statistically evaluating and testing consistency of multiple measurements of the same measurand presuming a randomly disturbed measurement process. Symbols: *Y* – (joint) measurand, *X_i_* – indicated quantities, *ξ* – possible values of the quantities *X_i_*, ***q***_1_,…,***q****_n_* – vectors of the repeated observations *q_ij_* where ***q****_i_* = [*q_i_*_1_, …,*q_ik_*]^T^, *g_Xi_* (*ξ*|***q****_i_*) – pdf for the quantity *X_i_* given the data ***q****_i_*, *h_Xi_* (***q****_i_*) – frequency distribution of the data ***q****_i_*.

Since the era of error analysis, metrologists have used the Birge chi-square test of statistical consistency to decide whether the differences between two or more measured values *x*_1_, …, *x_n_* are insignificant (Fig. 1). The Birge test is based on regarding the measured values *x*_1_, …, *x_n_* as realizations of random variables drawn from normal (Gaussian) sampling pdfs with unknown but equal expected values and known standard deviations [3]. When the measured values are correlated they are regarded as realizations of a random vector drawn from a joint *n*-variate normal distribution with a known variance-covariance matrix, referred to a normal consistency model. To assess statistical consistency of a set of measured values *x*_1_, …, *x_n_*, a common practice is to pretend that the standard uncertainties *u*(*x*_1_), …, *u*(*x_n_*) are the known standard deviations of the presumed normal sampling pdfs of *x*_1_, …, *x_n_*. It has previously been pointed out [4] that the Birge test and the concept of statistical consistency motivated by it do not apply to the results of measurement based on the GUM.

**Fig. 2:**
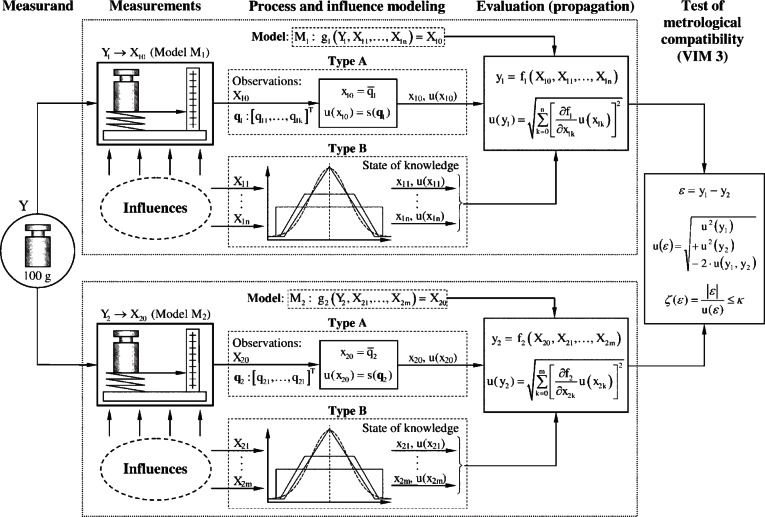
Illustration of an uncertainty approach to the metrological evaluation and test of compatibility of two measurements of the same measurand taking all known influences on the measurement processes into consideration. Symbols: *Y* – (joint) measurand, *Y*_1_, *Y*_2_ – measurand of the measurements, *X*_10_, *X*_20_, – indicated quantities, ***q***_1_,***q***_2_ – vectors of the repeated observations *q_ij_* where ***q****_i_* = [*q_i_*_1_, …, *q_ik_*]^T^, *X*_1_*_i_*, *X*_2_*_i_* – influence quantities with state of knowledge distributions. The elements in the grey blocks have been introduced by the GUM.

Recently, the VIM3 [2] introduced the idea of metro-logical compatibility, which can be used to assess the significance of the differences between two or more results of measurement for the same measurand (Fig. 2). As noted in [4] the concept of metrological compatibility fits with the GUM and it can be used to assess the significance of differences between results based on the GUM for the same measurand. In Sec. 2, we discuss the VIM3 definition of metrological compatibility and its consequences in more detail than done in [4]. In this paper we propose an approach for determining a combined result which is metrologically compatible with the contributing results whether or not the results as available were compatible. When a set of results for the same measurand turn out to be incompatible, the seemingly anomalous results must be investigated. In Sec. 3, we discuss the importance of documenting information which may be needed in such investigations. Sometimes multiple evaluations of the same measurand need to be combined. A legitimate combined result must be metrologically compatible with the contributing results. In Sec. 4, we propose an approach for determining a combined result which is metrologically compatible with the contributing results. In Sec. 5, we illustrate the proposed approach using published data from an interlaboratory evaluation of the same measurand. A brief summary is given in Sec. 6.

## 2. The VIM3 Concept of Metrological Compatibility

Generally, the measurand (quantity intended to be measured) is a property of a material or of a phenomenon. In many scientific, industrial, and commercial measurements, the measurand is sufficiently stable between multiple evaluations. Our primary interest is in such applications. Suppose two or more measurement procedures are used to measure the same measur-and. The measurement procedures may be (i) applications of the same method of measurement at different times or (ii) different implementations of a given method in different places or (iii) different methods.

A measured quantity value is a number together with a metrological reference (unit of measurement) expressing the magnitude of the quantity [2, Secs. 1.19 and 2.10] relative to the reference. A measured value must be traceable to a recognized metrological reference for it to be widely communicable. According to VIM3, two or more results of measurement for the same measurand are metrologically comparable if they are metrologically traceable to the same metrological reference [2, Sec. 2.46]. Metrological comparability does not imply that the measured values have similar magnitudes. The VIM3 concept of metrological compatibility applies only to those results of measurement which are metrologically comparable.

We assume that all results [*x*_1_, *u*(*x*_1_)], …, [*x_n_*, *u*(*x_n_*)] for a common measurand are traceable to the same metrological reference and hence they are metrologically comparable. Following the GUM [1], we use the symbol *X_i_* for a variable with a state-of-knowledge pdf represented by the result [*x_i_*, *u*(*x_i_*)], for *i* = 1, 2, …, *n*. The measured value *x_i_* is regarded as the expected value *E*(*X_i_*) and the standard uncertainty *u*(*x_i_*) is regarded as the standard deviation *S*(*X_i_*) of the pdf of *X_i_* for *i* = 1, 2, …, *n*. In the mainstream GUM, the pdf of *X_i_* is incompletely determined; the only thing reliably known about the pdf of *X_i_* is the expected value *E*(*X_i_*) = *x_i_* and the standard deviation *S*(*X_i_*) = *u*(*x_i_*), for *i* = 1, 2, …, *n*.

### 2.1 Metrological Compatibility of Two Particular Results

Metrological compatibility is defined for two results at a time. In the mainstream GUM, the difference *X*_1_ − *X*_2_ is a variable with an incompletely determined state-of-knowledge pdf for the difference between the values attributed by the two results [*x*_1_, *u*(*x*_1_)] and [*x*_2_, *u*(*x*_2_)] to the common measurand. The expected value and the standard deviation of the pdf of *X*_1_ − *X*_2_ are, respectively, *E*(*X*_1_ − *X*_2_) = *x*_1_ − *x*_2_ and *S*(*X*_1_ − *X*_2_) = √[*u*^2^(*x*_1_) + *u*^2^(*x*_2_) − 2*r*(*x*_1_, *x*_2_)*u*(*x*_1_)*u*(*x*_2_)], where *r*(*x*_1_, *x*_2_) is the correlation coefficient between *X*_1_ and *X*_2_. Following the GUM, we use the symbol *u*(*x*_1_ − *x*_2_) for the standard deviation *S*(*X*_1_ − *X*_2_).

According to the VIM3 [2, Sec. 2.47], two metrologically comparable results [*x*_1_, *u*(*x*_1_)] and [*x*_2_, *u*(*x*_2_)] for a measurand, supposed to be stable, are metrologically compatible if |*x*_1_ − *x*_2_| ≤ *κ* × *u*(*x*_1_ − *x*_2_) for a chosen threshold *κ*. According to the VIM3 [2, Sec. 2.47, Note 1], if two measurements for a common measurand, thought to be constant, are not metrologically compatible then there are two possibilities: (i) one or both of the measurements are incorrect (e.g., one or both of the measurement uncertainties are assessed as being too small) or (ii) the measurand changed between measurements.

We can use the VIM3 concept of metrological compatibility as a criterion to assess the significance of the differences between metrologically comparable results of measurement for the same measurand. In the mainstream GUM, the state-of-knowledge pdf represented by a result [*x_i_*, *u*(*x_i_*)], for *i* = 1, 2, …, *n*, is incompletely determined. Therefore, we need a quantitative measure for the difference between two fixed known results [*x*_1_, *u*(*x*_1_)] and [*x*_2_, *u*(*x*_2_)], each consisting of a measured value with standard uncertainty. Let us define a *ζ*-function, denoted by *ζ* (Δ), as

(1) $$\zeta (\Delta )=\frac{\left|\Delta \right|}{u(\Delta )}.$$

The value *ζ* (Δ) is a measure for the significance of the difference Δ. Even when a complete state-of-knowledge pdf of Δ is assumed, the metric (1) can be used to judge on the significance of the difference. Based on this metric we can restate the VIM3 definition of metrological comparability as follows [4]:

*Definition*: Two metrologically comparable results [*x*_1_, *u*(*x*_1_)] and [*x*_2_, *u*(*x*_2_)] for the same measurand are said to be metrologically compatible if

(2) $$\zeta (x_{1}-x_{2})=\frac{\left|x_{1}-x_{2}\right|}{u(x_{1}-x_{2})}\leq \kappa ,$$

for a chosen value of some threshold *κ*, where

(3) $$u(x_{1}-x_{2})=\sqrt{u^{2}(x_{1})+u^{2}(x_{2})-2r(x_{1},x_{2})u(x_{1})u(x_{2})},$$

and *r*(*x*_1_, *x*_2_) is the correlation coefficient between the variables *X*_1_ and *X*_2_ with state-of-knowledge pdfs represented by the results [*x*_1_, *u*(*x*_1_)] and [*x*_2_, *u*(*x*_2_)].

In definition 1, the value of *κ* is a chosen threshold for declaring metrological compatibility (lack of significant difference) of two results. Values for *ζ* (*x*_1_−*x*_2_) larger than *κ* are regarded as significant. The results are compatible, when the difference between the measured values *x*_1_ and *x*_2_ is insignificant in view of the standard uncertainties *u*(*x*_1_) and *u*(*x*_2_).

The VIM3 does not discuss how the threshold *κ* should be determined. A proper choice of the threshold *κ* is to a large extent a matter of agreement because it requires accepting the economic consequences of that choice. A conventional value of the threshold *κ* in metrology is two.

If one would agree on a larger value for *κ* then small differences are not detectable any more. This would be a disadvantage for applications when detecting small differences is important. But if we would agree on a smaller value for *κ* then a lot of small differences become significant even though they might be only a consequence of noisy measurements and the economic consequences are suffered by the metrological community trying to provide compatible measurement systems.

### 2.2 Metrological Compatibility of a Set of Results

According to the VIM3 [2, Sec. 2.47], a set of comparable results [*x*_1_, *u*(*x*_1_)], …, [*x_n_*, *u*(*x_n_*)], where *n* ≥ 2, is metrologically compatible if every one of the *n*(*n* − 1)/2 pairs of results [*x_i_*, *u*(*x_i_*)] and [*x_j_*, *u*(*x_j_*)], for *i*, *j* = 1, 2, …, *n* and *i* < *j*, is metrologically compatible. We can use expression (2) in this case by replacing *x*_1_ with *x_i_* and *x*_2_ with *x_j_*.

If for all pairs of results the values of *ζ*(*x_i_* − *x_j_*) are smaller than or equal to a chosen threshold *κ* then the set of results [*x*_1_, *u*(*x*_1_)], [*x*_2_, *u*(*x*_2_)], …, [*x_n_*, *u*(*x_n_*)] is metrologically compatible.

We can say that the differences between the measured values *x*_1_, …, *x_n_* are insignificant in view of the uncertainties *u*(*x*_1_), …, *u*(*x_n_*).

*Note* 1: A conventional idea that if the number *n* of the measured values *x*_1_, …, *x_n_* is large, it is natural to expect one or more of them to be significantly different from the rest comes from the theory of sampling from probability distributions having long tails which extend, for example, beyond two standard deviations. If the measurement procedures are properly carried out and the results of measurement are properly evaluated according to the GUM taking into account all important influence quantities, then a set of results for the same measurand should be metrologically compatible. When some results of measurement seem anomalous, they require explanation rather than acceptance. Often, anomalous results are consequence of missing important influence quantities.

### 2.3 Metrological Compatibility With a Reference Result

Suppose that in addition to the *n* measurement procedures, which yield the comparable results [*x*_1_, *u*(*x*_1_)], [*x*_2_, *u*(*x*_2_)], …, [*x_n_*, *u*(*x_n_*)], where *n* ≥ 2, the same measurand is measured by a higher echelon measurement procedure (or laboratory) yielding the reference result [*x*_R_, *u*(*x*_R_)], where *x*_R_ is the reference value with standard uncertainty *u*(*x*_R_). Alternatively, the common measurand may be a certified reference material of reference value *x*_R_ with standard uncertainty *u*(*x*_R_), which are not revealed before all *n* results of measurement are reported. We will use the symbol *X*_R_ for a variable with a state-of-knowledge pdf represented by the result [*x*_R_, *u*(*x*_R_)]. In general, the uncertainty *u*(*x*_R_) associated with the reference value *x*_R_ is smaller than the uncertainties *u*(*x*_1_), …, *u*(*x_n_*) associated with the measured values *x*_1_, …, *x_n_*.

If for all differences between the results *x_i_* and value *x*_R_, the values *ζ* (*x_i_* − *x*_R_) are smaller than or equal to a chosen threshold *κ* then the set of results [*x*_1_, *u*(*x*_1_)], [*x*_2_, *u*(*x*_2)_], …, [*x_n_*, *u*(*x_n_*)] is metrologically compatible with the reference value *x*_R_. We can say that the differences between the measured values *x*_1_, …, *x_n_* and the reference value *x*_R_ are insignificant in view of the uncertainties *u*(*x*_1_), …, *u*(*x_n_*) and *u*(*x*_R_).

One should not confuse the difference *ζ* (*x_i_* − *x*_R_) between the results [*x_i_*, *u*(*x_i_*)] and [*x*_R_, *u*(*x*_R_)] with *E_n_*-values which do not seem to be uniquely defined.^1^

### 2.4 Metrological Compatibility With a Combined Result

Sometimes the results [*x*_1_, *u*(*x*_1_)], [*x*_2_, *u*(*x*_2_)], …, [*x_n_*, *u*(x*_n_*)], where *n* ≥ 2, need to be combined to determine a combined result [*x*_C_, *u*(*x*_C_)], where *x*_C_ is the combined value and *u*(*x*_C_) is the standard uncertainty associated with *x*_C_. We will use the symbol *X*_C_ for a variable with a state-of-knowledge pdf represented by [*x*_C_, *u*(*x*_C_)]. In accordance with the GUM, the combined variable *X*_C_ for a value of the measurand should be defined as a measurement function of the input variables *X*_1_, …, *X_n_*. Often, *X*_C_ is set as a convex linear combination of *X*_1_, …, *X_n_* with non-negative weights *a*_1_, …, *a_n_* which sum up to one. Thus often a measurement function for *X*_C_ is of the form

(4) $$X_{C}=\sum {}_{i}a_{i}X_{i},$$

where *a_i_* ≥ 0 and Σ*_i_a_i_* = 1, for *i* = 1, 2, …, *n*. Since (4) is a linear function in *X_i_* the expected value *E*(*X*_C_) of *X*_C_ is the combined value *x*_C_, where

(5) $$X_{C}=\sum {}_{i}a_{i}x_{i},$$

and the standard deviation *S*(*X*_C_) of *X*_C_ is the standard uncertainty *u*(*x*_C_) where

(6) $$\begin{array}{l} u^{2}(x_{C})=\sum {}_{i}a_{i}^{2}u^{2}(x_{i})\\ \;+2\sum {}_{i<j}a_{i}a_{j}u(x_{i})u(x_{j})r(x_{i},x_{j}). \end{array}$$

If the individual measurement procedures are all uncorrelated then the cross-product term in (6) is zero.

If *a_i_* = 1/*n* for *i* = 1, 2, …, *n*, then *X*_C_ reduces to the arithmetic average *X*_A_ = (1/*n*) Σ*_i_ X_i_*. The expected value *E*(*X*_A_) is *x*_A_ = (1/*n*) Σ*_i_ x_i_* and the standard deviation *S*(*X*_A_) denoted by *u*(*x*_A_) can be determined from (6). If the pdfs for *X*_1_, …, *X_n_* are uncorrelated, then

(7) $$u^{2}(x_{A})=\frac{1}{n^{2}}\sum_{i}u^{2}(x_{i}).$$

If *a_i_* = *w_i_*/Σ*_i_ w_i_*, where *w_i_* = 1/*u*^2^(*x_i_*) then *X*_C_ reduces to the weighted mean *X*_W_ = Σ*_i_ w_i_ X_i_*/Σ*_i_ w_i_* with weights inversely proportional to the variances *u*^2^(*x*_1_), …, *u*^2^(*x_n_*). The expected value *E*(*X*_W_) is *x*_W_ = Σ*_i_ w_i_ x_i_*/Σ*_i_ w_i_* and the standard deviation *S*(*X*_W_) denoted by *u*(*x*_W_) can be determined from (6). If the pdfs for *X*_1_, …, *X_n_* are uncorrelated, then

(8) $$u^{2}(x_{W})=\frac{1}{\Sigma_{i}w_{i}}=\frac{1}{\Sigma_{i}(1/u^{2}(x_{i}))}.$$

If for all differences between the results *x_i_* and the combined value *x*_C_, the values *ζ* (*x_i_* − *x*_C_) are smaller than or equal to a chosen threshold *κ* then the set of results [*x*_1_, *u*(*x*_1_)], [*x*_2_, *u*(*x*_2_)], …, [*x_n_*, *u*(*x_n_*)] is metro-logically compatible with the combined value *x*_C_. Then we can say that the differences between the measured values *x*_1_, …, *x_n_* and the combined value *x*_C_ are insignificant in view of the uncertainties *u*(*x*_1_), …, *u*(*x_n_*).

In evaluating *u*(*x_i_* − *x*_C_) the correlation coefficient between *X_i_* and *X*_C_ must be included because the pdfs of *X_i_* and *X*_C_ are always correlated, for *i* = 1, 2, …, *n*. For example, if the pdfs for *X*_1_, …, *X_n_* are uncorrelated, then the variance, *V*(*X_i_* − *X*_C_), denoted by *u*^2^(*x_i_* − *x*_C_) is

(9) $$u^{2}(x_{i}-x_{C})=u^{2}(x_{i})+\sum_{i}a_{i}^{2}u^{2}(x_{i})-2a_{i}u^{2}(x_{i}).$$

If *a_i_* = 1/*n*, for *i* = 1, 2, …, *n*, then *x*_C_ reduces to the arithmetic average *x*_A_ = (1/*n*) Σ*_i_ x_i_* and the uncertainty *u*(*x_i_* − *x*_C_) given in (9) reduces to *u*(*x_i_* − *x*_A_), where

(10) $$u^{2}(x_{i}-x_{A})=\left(\frac{n-2}{n}\right)u^{2}(x_{i})+u^{2}(x_{A}).$$

If *a_i_* = *w_i_*/Σ*_i_ w_i_*, where *w_i_* = 1/*u*^2^(*x_i_*), for *i* = 1, 2, …, *n*, then *x*_C_ reduces to the weighted mean *x*_W_ = Σ*_i_ w_i_x_i_*/Σ*_i_ w_i_* and the uncertainty *u*(*x_i_* − *x*_C_) given in (9) reduces to *u*(*x_i_* − *x*_W_), where

(11) $$u^{2}(x_{i}-x_{W})=u^{2}(x_{i})-u^{2}(x_{W}).$$

If the uncertainties *u*(*x*_1_), *u*(*x*_2_), …, *u*(*x_n_*) were all equal to *u*(*x*), say, then *x*_W_ reduces to *x*_A_ and *u*^2^(*x*_W_) reduces to *u*^2^(*x*_A_) = *u*^2^(*x*)/*n*. Then both (10) and (11) reduce to

(12) $$u^{2}(x)-\frac{u^{2}(x)}{n}=\frac{n-1}{n}u^{2}(x).$$

*Note* 2: Sometimes, the standard uncertainties *u*(*x*_1_), *u*(*x*_2_), …, *u*(*x_n_*) are not all reliably determined. Also, the standard uncertainties are frequently inappropriate bases for assigning the weights *a*_1_, *a*_2_, …, *a_n_* to the measured values *x*_1_, *x*_2_, …, *x_n_* to determine a combined result. Therefore the weighted mean *x*_W_ may be inappropriate for combining the values. Thus, in our view, the arithmetic mean *x*_A_ should be regarded as a default combined value.

## 3. Information Needed to Determine Sources of Incompatibility

A purpose of assessing metrological compatibility is to demonstrate lack of significant difference between the results of measurement for a common measurand. If a set of results turns out to be metrologically incompatible then the measurement procedures and calculations underlying the seemingly anomalous results should be investigated. Every result of measurement should have supporting documents which include the measurement function (measurement equation) and complete uncertainty budget. If the influence quantities, uncertainty components, and correlation coefficients identified in the uncertainty budget are reasonable then in search of the possible sources of incompatibility one must look into potential influence quantities not included in the uncertainty budget.

Investigations to determine the sources of incompatibility are generally done in retrospect long after completing the measurements. Therefore investigators need detailed descriptions of what was actually done during measurement. Often, metrologists do not have enough time and resources to document in sufficient detail for retrospective investigation what was actually done in a particular application of the measurement procedure. In the absence of such documentation it may be difficult to determine possible sources of incompatibility.

*Note* 3: We hope that in the not too distant future, metrologists and information technology experts would collaborate to develop tools which make it easier for metrologists to document in real time the actual measurement procedure while the measurements are being done. Such documentation should be helpful in identifying all potentially important influence quantities.

## 4. Determination of a Combined Value and Its Associated Uncertainty

Even when the common measurand is sufficiently stable, the results [*x*_1_, *u*(*x*_1_)], …, [*x_n_*, *u*(*x_n_*)] can exhibit large variation. Metrological incompatibility occurs when some or all results (measured values or standard uncertainties) are improperly determined. Frequently, improper results are consequence of missing important influence quantities. For example, in many chemical measurements, the measurand is the amount of one component in a sample of multi-component material. The other components can interfere with the measurements. Frequently, it is impossible to know all potential interferences. Therefore, it is difficult to be sure that all significant influence quantities have been accounted for in determining the measured values and uncertainties.

For a combined result [*x*_C_, *u*(*x*_C_)] to be legitimate it should be metrologically compatible with the contributing results of measurement [*x*_1_, *u*(*x*_1_)], …, [*x_n_*, *u*(*x_n_*)]. Therefore we propose the following principle.

*Principle for combining multiple results for the same measurand*: Determine the combined result [*x*_C_, *u*(*x*_C_)] from the expressions (5) and (6) as recommended in the GUM. If the results [*x*_1_, *u*(*x*_1_)], [*x*_2_, *u*(*x*_2_)], …, [*x_n_*, *u*(*x_n_*)] are metrologically compatible with the combined result [*x*_C_, *u*(*x*_C_)], then *u*(*x*_C_) is a valid expression for the standard uncertainty associated with *x*_C_. If the results [*x*_1_, *u*(*x*_1_)], [*x*_2_, *u*(*x*_2_)], …, [*x_n_*, *u*(*x_n_*)] are metrologically incompatible with the combined result [*x*_C_, *u*(*x*_C_)], then the seemingly anomalous results should be investigated. Until the investigation resolves the anomalous results, in the absence of additional knowledge, all results in a metrologically incompatible set should be regarded with suspicion. To determine a legitimate combined result, we propose that the measured values *x*_1_, …, *x_n_* should be sustained and each of the uncertainties *u*(*x*_1_), *u*(*x*_2_), …, *u*(*x_n_*) should be enlarged just enough to make the results [*x*_1_, *u*(*x*_1_)], [*x*_2_, *u*(*x*_2_)], …, [*x_n_*, *u*(*x_n_*)] metrologically compatible with the combined result [*x*_C_, *u*(*x*_C_)].

This approach was first proposed in [5] and has recently been used in [6]. Thus we define variables *Y*_1_, …, *Y_n_* with corrected state-of-knowledge pdfs for the common measurand as follows

(13) $$Y_{i}=X_{i}+\delta X_{i},$$

where *δX*_1_, …, *δX_n_* are correction variables. Then a measurement function for the combined variable *Y*_C_ is

(14) $$\begin{array}{c} \;Y_{C}=\sum_{i}a_{i}Y_{i}=\sum_{i}a_{i}X_{i}+\sum_{i}a_{i}\delta X_{i}\\ =X_{C}+\sum_{i}a_{i}\delta X_{i}, \end{array}$$

where *a_i_* ≥ 0 and Σ*_i_ a_i_* = 1, for *i* = 1, 2, …, *n*, and the pdfs for the correction variables *δX*_1_, …, *δX_n_* are mutually independent and independent of the pdfs for *X*_1_, …, *X_n_*. The pdfs assigned to the correction variables *δX*_1_, …, *δX_n_* express the limits of knowledge. Thus, we assign zero expected values and the same variance *u*^2^(*δ*) to each of the correction variables *δX*_1_, …, *δX_n_*. Thus the expected value *E*(*δX_i_*) is zero and the variance *V*(*δX_i_*) is *u*^2^(*δ*), for *i* = 1, 2, …, *n*. It follows from (13) that the expected value *y_i_* and the variance *u*^2^(*y_i_*) of the pdf for *Y_i_* are

(15) $$y_{i}=x_{i}+0=x_{i},$$

and

(16) $$u^{2}(y_{i})=u^{2}(x_{i})+u^{2}(\delta ),$$

for *i* = 1, 2, …, *n*.

We propose that the variance *u*^2^(*δ*) should be set just large enough to make the results [*y*_1_, *u*(*y*_1_)], [*y*_2_, *u*(*y*_2_)], …, [*y_n_*, *u*(*y_n_*)] compatible with the result [*y*_C_, *u*(*y*_C_)]. As discussed in Sec. 2.4, the results [*y*_1_, *u*(*y*_1_)], [*y*_2_, *u*(*y*_2_)], …, [*y_n_*, *u*(*y_n_*)] are compatible with [*y*_C_, *u*(*y*_C_)] when

(17) $$\zeta (y_{i}-y_{C})=\frac{\left|y_{i}-y_{C}\right|}{u(y_{i}-y_{C})}\leq \kappa ,$$

or equivalently

(18) $${\left(y_{i}-y_{C}\right)}^{2}\leq \kappa^{2}\times u^{2}(y_{i}-y_{C}),$$

for all *i* = 1, 2, …, *n*. From (15), we have

(19) $$y_{C}=\sum_{i}a_{i}y_{i}=\sum_{i}a_{i}x_{i}=x_{C},$$

and

(20) $$y_{i}-y_{C}=x_{i}-x_{C}.$$

From the appendix, we have

(21) $$u^{2}(y_{i}-y_{c})=u^{2}(x_{i}-x_{c})+u^{2}(\delta )[1+{\sum {}_{i}a_{i}}^{2}-2a_{i}].$$

Therefore, the criterion of compatibility (18) is equivalent to

(22) $${\left(x_{i}-x_{C}\right)}^{2}\leq \kappa^{2}\times \left(u^{2}(x_{i}-x_{C})+u^{2}(\delta )[1+\sum {}_{i}a_{i}^{2}-2a_{i}]\right),$$

for all *i* = 1, 2, …, *n*. It follows that

(23) $$u^{2}(\delta )\geq \frac{1}{[1+\sum {}_{i}a_{i}^{2}-2a_{i}]}\left(\frac{{\left(x_{i}-x_{C}\right)}^{2}}{\kappa^{2}}-u^{2}(x_{i}-x_{C})\right),$$

for all *i* = 1, 2, …, *n*. Thus, if *u*^2^(*δ*) is chosen as

(24) $$\begin{array}{l} u^{2}(\delta )=\max(0,\{\frac{1}{[1+\sum {}_{i}a_{i}^{2}-2a_{i}]}\\ \;\left(\frac{{\left(x_{i}-x_{C}\right)}^{2}}{\kappa^{2}}-u^{2}(x_{i}-x_{C})\right)|i=1,\ldots ,n\}), \end{array}$$

then each of the corrected measured values *y*_1_, …, *y_n_* would be metrologically compatible with the combined measured value *y*_C_. If the measured values *x*_1_, …, *x_n_* are compatible with the combined measured value *x*_C_ then each of the *n* quantities in the curly parenthesis of (24) are negative and *u*^2^(*δ*) = 0. In that case the measurement function (14) reduces to (5) and the uncertainty associated with the combined measured value *x*_C_ is given by (6).

### 4.1 Arithmetic Average

If *a_i_* = 1/*n*, for *i* = 1, 2, …, *n*, then *x*_C_ reduces to the arithmetic average *x*_A_ and from (24),

(25) $$\begin{array}{l} u^{2}(\delta )=\max(0,\{\frac{n}{n-1}\left(\frac{{\left(x_{i}-x_{A}\right)}^{2}}{\kappa^{2}}-u^{2}(x_{i}-x_{A})\right)\\ \;\;|i=1,\ldots ,n\}). \end{array}$$

The combined value *y*_C_ reduces to *y*_A_ = (1/*n*) Σ*_i_ y_i_* = (1/*n*) Σ*_i_ x_i_* = *x*_A_. To assure that the measured values *y*_1_, …, *y_n_* are compatible with *y*_A_ one can check that

(26) $$\zeta (y_{i}-y_{A})=\frac{\left|y_{i}-y_{A}\right|}{u(y_{i}-y_{A})}\leq \kappa ,$$

where as shown in the appendix

(27) $$u^{2}(y_{i}-y_{A})=u^{2}(x_{i}-x_{A})+\frac{n-1}{n}u^{2}(\delta ).$$

Expressions for *u*^2^(*x_i_*− *x*_A_) and *u*^2^(*δ*) are given in (10) and (25), respectively. The uncertainty associated with *y*_A_ is from (7)

(28) $$u^{2}(y_{A})=\frac{1}{n^{2}}\sum_{i}u^{2}(y_{i})=\frac{1}{n^{2}}\sum_{i}\left(u^{2}(x_{i})+u^{2}(\delta )\right).$$

If *u*^2^(*δ*) = 0, then (28) reduces to (7).

### 4.2 Weighted Mean

Since the variance associated with *y_i_* is *u*^2^(*y_i_*) = *u*^2^(*x_i_*) + *u*^2^(*δ*), a weighted mean with weights inversely proportional to the variances of the results *y*_1_, …, *y_n_* is *y*_W_ = Σ*_i_ w_i_ y_i_*/Σ*_i_ w_i_*, where *y_i_* = *x_i_*, and *w_i_* = 1/*u*^2^(*y_i_*) = 1/[*u*^2^(*x_i_*) + *u*^2^(*δ*)] for *i* = 1, 2, …, *n*. The measured values *y*_1_, …, *y_n_* are compatible with *y*_W_ if

(29) $$\zeta (y_{i}-y_{W})=\frac{\left|y_{i}-y_{W}\right|}{u(y_{i}-y_{W})}\leq \kappa ,$$

for all *i* = 1, 2, …, *n*. Analogous to (11)

(30) $$u^{2}(y_{i}-y_{W})=u^{2}(y_{i})-u^{2}(y_{W}),$$

where

(31) $$u^{2}(y_{W})=\frac{1}{\sum_{i}\left(1/[u^{2}(x_{i})+u^{2}(\delta )]\right)}.$$

The variance *u*^2^(*δ*) is the smallest value which would make the measured values *y*_1_, …, *y_n_* compatible with *y*_W_. Such a value for *u*^2^(*δ*) can be iteratively determined using the value of *u*^2^(*δ*) from (25) as a starting value.

*Note* 4: Let us use the symbol *Y*_true_ for a true quantity value [2, Sec. 2.11] of the common measurand commensurate with its description. (In the GUM, the same symbol *Y* is also used for a quantity with a state-of-knowledge pdf for the common measurand.) If the measurand is defined in extensive detail, a true value *Y*_true_ may be essentially unique. If the measurand is defined in less detail, then a range of values may be commensurate with its definition and any one of them qualifies as a true value *Y*_true_ of the measurand. The concept of metrological compatibility relates to the observed differences between the measured values *x*_1_, …, *x_n_* rather than to the unobservable differences between the measured values and a true value *Y*_true_ of the measurand. Therefore, regardless of whether the measured values *x*_1_, …, *x_n_* are compatible or incompatible with the combined value *x*_C_, the measured values alone provide no information about the difference between *x*_C_ and *Y*_true_. In particular, metrological compatibility does not imply that the difference between *x*_C_ and *Y*_true_ is not significant. However, there is no factual knowledge about potential significant difference between *x*_C_ and *Y*_true_. Therefore, a correction applied to *x*_C_ for its potential significant difference between *x*_C_ and *Y*_true_ and enlargement of the uncertainty *u*(*x*_C_) determined from (6) as discussed in [7] would be arbitrary.

## 5. Combined Result From an Interlaboratory Evaluation

The Columns 2 and 3 of table 1 reproduce from [8, Table 3] the measured values, *c*_Lab_, and the corresponding standard uncertainties, *u*(*c*_Lab_), for the amount content of lead (Pb) in natural river water as determined by the eight laboratories^2^ identified in column 1 of table 1. We will use these data to illustrate calculation of a combined result. Suppose the arithmetic average *c*_Avg_ = 62.79 nmol/kg is used as the combined measured value. The associated standard uncertainty based on the expression (7) is *u*(*c*_Avg_) = 0.26 nmol/kg. The values of *ζ*(*c*_Lab_ − *c*_Avg_) between the reported results [*c*_Lab_, *u*(*c*_Lab_)] and the combined result [*c*_Avg_, *u*(*c*_Avg_)] determined by using the expression (10) for the standard uncertainty *u*(*c*_Lab_ − *c*_Avg_) are shown in column 4 of table 1. Suppose the threshold for metrological compatibility is set as *κ* = 2. One of the values of *ζ*(*c*_Lab_ − *c*_Avg_) (from LNE) is larger than 2.00. Therefore not all of the eight reported results [*c*_Lab_, *u*(*c*_Lab_)] are metrologically compatible with the combined result [*c*_Avg_, *u*(*c*_Avg_)]. Until potential flaws in the deviant result (from LNE or the others) are determined, all results must be regarded with suspicion. Therefore, as discussed in Sec. 4, we propose that all reported measured values should be sustained and each of the uncertainties should be enlarged by the amount *u*^2^(*δ*) = 1.130 determined from the expression (25). The adjusted (enlarged) standard uncertainties *u*(*c*_Lab_) based on the expression (16) are shown in column 5 of table 1. Based on the adjusted uncertainties *u*(*c*_Lab_), the standard uncertainty associated with the arithmetic mean *c*_Avg_ determined from the expression (28) is *u*(*c*_Avg_) = 0.46 nmol/kg. The differences *ζ*(*c*_Lab_, *c*_Avg_) based on the adjusted uncertainties are shown in column 6 of table 1. Since none of the values of *ζ*(*c*_Lab_ − *c*_Avg_) is larger than 2.00, the adjusted results [*c*_Lab_, *u*(*c*_Lab_)] given in columns 2 and 5 of table 1 are metrologically compatible with the combined result [*c*_Avg_, *u*(*c*_Avg_)].

**Fig. 3:**
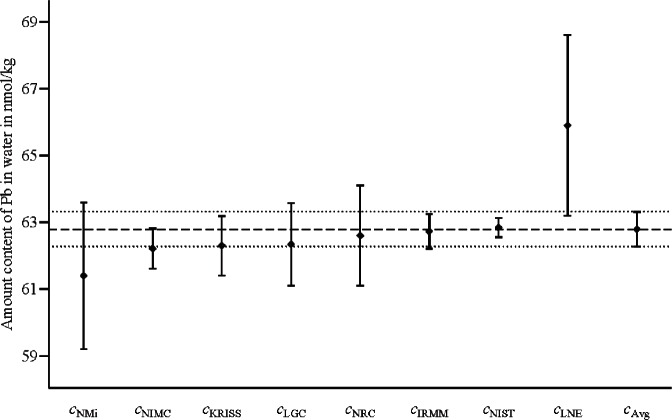
The measured values *c*_Lab_ and their arithmetic average *c*_Avg_ for the amount content of lead (Pb) with the expanded uncertainty intervals (for coverage factor *k* = 2) determined from the uncertainties stated in the report [8] and reproduced in column 3 of table 1. The arithmetic average is *c*_Avg_ = 62.79 nmol/kg with standard uncertainty *u*(*c*_Avg_) = 0.26 nmol/kg.

**Fig. 4:**
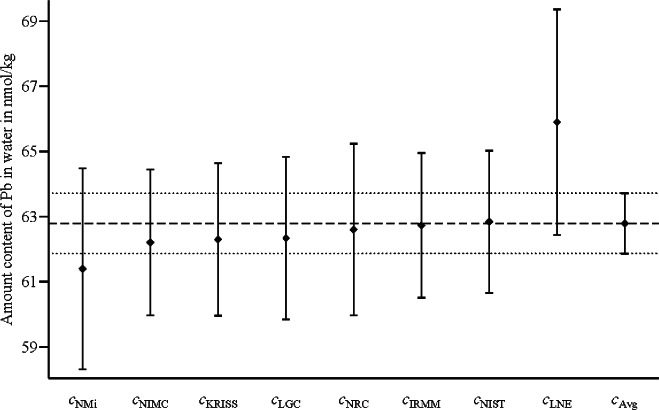
The measured values *c*_Lab_ and their arithmetic average *c*_Avg_ for the amount content of lead (Pb) with the expanded uncertainty intervals (for coverage factor *k* = 2) determined from the adjusted (enlarged) uncertainties given in column 5 of table 1. The arithmetic average is *c*_Avg_ = 62.79 nmol/kg with standard uncertainty *u*(*c*_Avg_) = 0.46 nmol/kg.

Figures 3 and 4 display the measured values *c*_Lab_ (given in column 2 of table 1) and the arithmetic average *c*_Avg_ = 62.79 nmol/kg along with the corresponding expanded uncertainty intervals (for coverage factor *k* =2). In Fig. 3, the expanded uncertainty intervals are based on the standard uncertainties as reported in [8] and reproduced in column 3 of table 1; in particular, the standard uncertainty *u*(*c*_Avg_) associated with *c*_Avg_ is *u*(*c*_Avg_) = 0.26 nmol/kg. In Fig. 4, the expanded uncertainty intervals are based on the adjusted (enlarged) standard uncertainties displayed in column 5 of table 1; in particular, the standard uncertainty *u*(*c*_Avg_) associated with *c*_Avg_ is *u*(*c*_Avg_) = 0.46 nmol/kg.

In both Figs. 3 and 4, the expanded uncertainty intervals (for coverage factor *k* = 2) for the measured values overlap with the expanded uncertainty interval for the arithmetic average *c*_Avg_. However, not all of the eight results in Fig. 3 are metrologically compatible with the combined result [*c*_Avg_, *u*(*c*_Avg_)]. This shows that there is no direct correspondence between the overlap of the expanded uncertainty intervals (for coverage factor *k* = 2) and the VIM3 concept of metrological compatibility.

## 6. Summary

The VIM3 [2] concept of metrological compatibility applies to only those results which are metrologically comparable; that is, the results must be traceable to the same reference. Metrological compatibility is a pairwise concept. Two metrologically comparable results for the same measurand are said to be metrologically compatible if the ζ-value of the difference between the results is less than or equal to a chosen threshold (usually 2.0). A set of metrologically comparable results is metrologically compatible if all of the distinct pairs of results are metrologically compatible. The concept of metrological compatibility easily extends to compatibility of a set of results with a reference result or a combined result. Metrological compatibility does not require complete knowledge of the pdfs represented by the results of measurement.

Often multiple evaluations for the same measurand must be combined to determine a combined result. For a combined result to be legitimate it should be metrologically compatible with the contributing results of measurement. When the results are metrologically incompatible with the combined result, we propose that the measured values should be sustained and each of the standard uncertainties should be enlarged just enough to make the results compatible with the combined result. Then the results can be combined using the GUM. This approach has been found to be useful in many practical applications.

## Figures and Tables

**Table 1:** The measured values *c*_Lab_ for the amount content of Pb in natural river water and their associated standard uncertainties *u*(*c*_Lab_) in nmol/kg units as reported in [8]. Also shown are the differences *ζ*(*c*_Lab_ − *c*_Avg_) based on the reported uncertainties and the adjusted (enlarged) uncertainties

Laboratory Identifier	Amount Content *c*_Lab_/nmol/kg	Reported Uncertainty *u*(*c*_Lab_)/nmol/kg	Reported *ζ*-value	Adjusted Uncertainty *u*(*c*_Lab_)/nmol/kg	Adjusted *ζ*-value
NMi	61.40	1.10	1.40	1.53	0.99
NIMC	62.21	0.30	1.56	1.10	0.54
KRISS	62.30	0.45	1.04	1.15	0.44
LGC	62.34	0.62	0.75	1.23	0.38
NRC	62.60	0.75	0.27	1.30	0.15
IRMM	62.70	0.26	0.25	1.09	0.08
NIST	62.84	0.15	0.19	1.07	0.05
LNE	65.90	1.35	2.60	1.72	2.00

## Footnotes

1 One version defines *E_n_*-value as *E_n_* = (*x_i_* − *x*_R_)/√[(2*s_i_*)^2^ + (2*s*_R_)^2^], where *x_i_* and *x*_R_ are regarded as realizations of random variables with sampling pdfs and *s_i_* and *s*_R_ are the estimated standard deviations of those sampling pdfs. Thus *E_n_*-values are realizations of random variables with sampling pdfs. Some metrologists substitute in the denominator of the *E_n_*-value, the expanded standard uncertainties for 2*s_i_* and 2*s*_R_. This is inappropriate uses of the expanded standard uncertainties.

2 Reference [8] is the final report of the CIPM international key comparison CCQM-K2. In this paper, we have used data from [8] to illustrate calculation of a combined result. We do not address data analysis of a key comparison to determine the key comparison reference value (KCRV) and the degrees of equivalence (DOE).

## 7. Appendix

Since *δX*_C_ = Σ*_i_a_i_δX_i_*, we have expected value *E*(*δX*_C_) = 0 and variance $V(\delta X_{C})=u^{2}(\delta )\sum_{i}a_{i}^{2}$. Thus $E(\delta X_{i}-\delta X_{C})=0\text{and}\;V(\delta X_{i}-\delta X_{C})=V(\delta X_{i})+V(\delta X_{C})-2C(\delta X_{i},\delta X_{C})=u^{2}(\delta )[1+\sum_{i}a_{i\cdot }^{2}-2a_{i}]$, where the third term is covariance. Therefore $u^{2}(y_{i}-y_{C})=V(Y_{i}-Y_{C})=V(X_{i}-X_{C})+V(\delta X_{i}-\delta X_{C})=u^{2}(x_{i}-x_{C})+u^{2}(\delta )[1+\sum_{i}a_{i\cdot }^{2}-2a_{i}]$.

If *a_i_* = 1/*n*, for *i* = 1, 2, …, *n*, then $u^{2}(y_{i}-y_{A})=u^{2}(x_{i}-x_{A})+\frac{n-1}{n}u^{2}(\delta )$.
